# Atypical hemolytic uremic syndrome in first trimester pregnancy successfully treated with eculizumab

**DOI:** 10.1186/s40164-017-0064-7

**Published:** 2017-01-13

**Authors:** Gabriela Andries, Michael Karass, Srikanth Yandrapalli, Katherine Linder, Delong Liu, John Nelson, Rahul Pawar, Savneek Chugh

**Affiliations:** 1 Division of Internal Medicine, New York Medical College, Westchester Medical Center, Valhalla, NY 10595 USA; 2 Division of Hematology and Oncology, New York Medical College, Westchester Medical Center, Valhalla, NY 10595 USA; 3 Division of Nephrology, New York Medical College, Westchester Medical Center, Valhalla, NY 10595 USA

**Keywords:** Thrombotic microangiopathy (TMA), Atypical hemolytic uremic syndrome (aHUS), Eculizumab, Pregnancy, First trimester

## Abstract

**Background:**

Atypical hemolytic uremic syndrome is a rare disorder which is known to cause acute thrombotic microangiopathy during pregnancy with poor maternal and fetal outcomes. Atypical hemolytic uremic syndrome is caused mostly by dysregulation of alternative complement pathway secondary to genetic mutations. Most of the cases reported have been in the post-partum period. We report a rare case of a patient who presents with thrombotic microangiopathy in the first trimester of her eleventh pregnancy and was successfully treated with eculizumab.

**Case presentation:**

A 30-year-old woman presented at 10 weeks of gestation with hypertension, hemolytic anemia, thrombocytopenia, and acute kidney injury, consistent with thrombotic microangiopathy. She was managed initially with daily plasmapheresis. However, her kidney function did not recover, requiring hemodialysis. ADAMTS13 activity was later found to be within normal limit, hence diagnosis of atypical hemolytic uremic syndrome was strongly considered at that time and she was immediately treated with anti-C5 humanized monoclonal antibody (eculizumab). The patient responded well (resolution of thrombotic microangiopathy and recovery of renal function) to eculizumab, with continued remission after discharge and successfully delivered a healthy baby at term without any peripartum complications.

**Conclusion:**

Early recognition of atypical hemolytic uremic syndrome is often difficult as several other conditions also manifest as thrombotic microangiopathy during pregnancy, causing delay in initiating appropriate treatment. Our case suggests that treatment of atypical hemolytic uremic syndrome in early trimester of pregnancy with eculizumab results in good outcome to mother and fetus.

## Background

Thrombotic microangiopathy (TMA) is a group of disorders with a common pathological pattern of injury characterized by fibrin and/or platelet thrombi in the microvasculature causing consumptive thrombocytopenia, hemolytic anemia and end organ damage. Diseases causing primary TMA are divided into two broad categories: thrombotic thrombocytopenic purpura (TTP) and hemolytic uremic syndrome (HUS). In TTP, systemic clumping of platelets is caused by unusually large multimers of von Willebrand factor as a consequence of deficient ADAMTS13 activity (a von Willebrand factor-cleaving metalloprotease). Hemolytic uremic syndrome (HUS), on the other hand, is not associated with the absence or severe reduction of ADAMTS13 activity and has predominant renal involvement.

HUS is divided into typical (shiga-toxin mediated or STEC) HUS and atypical HUS (aHUS) [1]. Atypical HUS occurs due to dysregulation of the alternative complement pathway, leading to complement-mediated endothelial damage resulting in TMA and organ injuries. Pregnancy has been associated with a wide spectrum of TMA, ranging from HUS, TTP, and HELLP (hemolysis, elevated liver enzymes, and low platelet count). Distinction among these three syndromes is often difficult as they share similar clinical features. Establishing the right diagnosis is very crucial for implementing the optimal treatment strategies [2]. We present a very rare case of a young lady who presented during the first trimester of pregnancy with severe thrombocytopenia, hemolytic anemia, and acute kidney injury (AKI). She was initially suspected to have TTP, but later diagnosed with an unusually early presentation of atypical HUS with dramatic clinical recovery and successful pregnancy outcome with the use of eculuzimab.

## Case report

A 30-year-old woman, presented to the hospital at 10 weeks of gestation, with nausea, vomiting, few episodes of watery non-bloody diarrhea, and dark colored urine for the past 5 days. She had multiple abortions in the past and was having her 11th pregnancy, all with the same partner. Her past medical history is significant for hereditary pancreatitis (R117 H gene mutation) requiring multiple surgeries, five miscarriages (all in the first trimester), and self-resolving thrombocytopenia during the last trimester of her previous viable pregnancies. She tested negative for antiphospholipid syndrome (negative anticardiolipin antibodies, anti-beta2-glycoprotein I antibodies, and lupus anticoagulant). She denied any bleeding or bruising symptoms including hematuria, epistaxis, or vaginal bleeding.

She presented with a blood pressure of 156/99 mmHg, heart rate of 80 beats/min, respiratory rate of 16 breaths/min, oral temperature of 98.4°F, and peripheral oxygen saturation of 97% on ambient air. Physical examination was unremarkable, except for petechiae on the lower and upper extremities. Initial laboratory investigations revealed a hemoglobin level of 7.8 mg/dL, platelet count of 15,000/mm^3^, presence of schistocytes on peripheral smear, serum creatinine of 2.44 mg/dL, elevated lactate dehydrogenase (LDH) at 1847 U/L, aspartate aminotransferase (AST) 58 U/L, alanine aminotransferase (ALT) 17 U/L, alkaline phosphatase 45 U/L, total bilirubin 1.7 mg/dL, direct bilirubin of 0.4 mg/dL, albumin 3.2 mg/dL, and haptoglobin <8 mg/dL.

Urinalysis showed 3+ proteinuria and 3+ blood with spot urine protein-to-creatinine ratio of 5600 mg/g. Her coagulation studies were normal with INR 0.95, PT 10.4 s, PTT 25.5 s and fibrinogen level of 311 mg/dL (Table 1).

**Table 1:** Patient’s laboratory result at initial presentation

Test	Patient’s result	Normal reference value
Hemoglobin (g/dL)	7.8	11.6–15
Hematocrit (%)	22.1	36–45
WBC (K/CU MM)	7.9	4.5–10.8
Platelet count (K/U MM)	15	160–410
BUN (mg/dL)	65	6–22
Creatinine (mg/dL)	2.44	0.57–1.11
LDH (U/L)	1847	125–220
AST (U/L)	58	4–35
ALT (U/L)	17	6–55
Total bilirubin (mg/dL)	1.7	0.2–1.3
Direct bilirubin (mg/dL)	0.4	0.1–0.6
Haptoglobin (mg/dL)	<8	13–281
Amylase level (U/L)	30	22–100
Lipase level (U/L)	8	8–78
Prothrombin time (s)	9.8	9.8–12
INR	0.95	
Partial thromboplastin time (s)	27.5	25–32
Fibrinogen level (mg/dL)	432	180–400

**Fig. 1:**
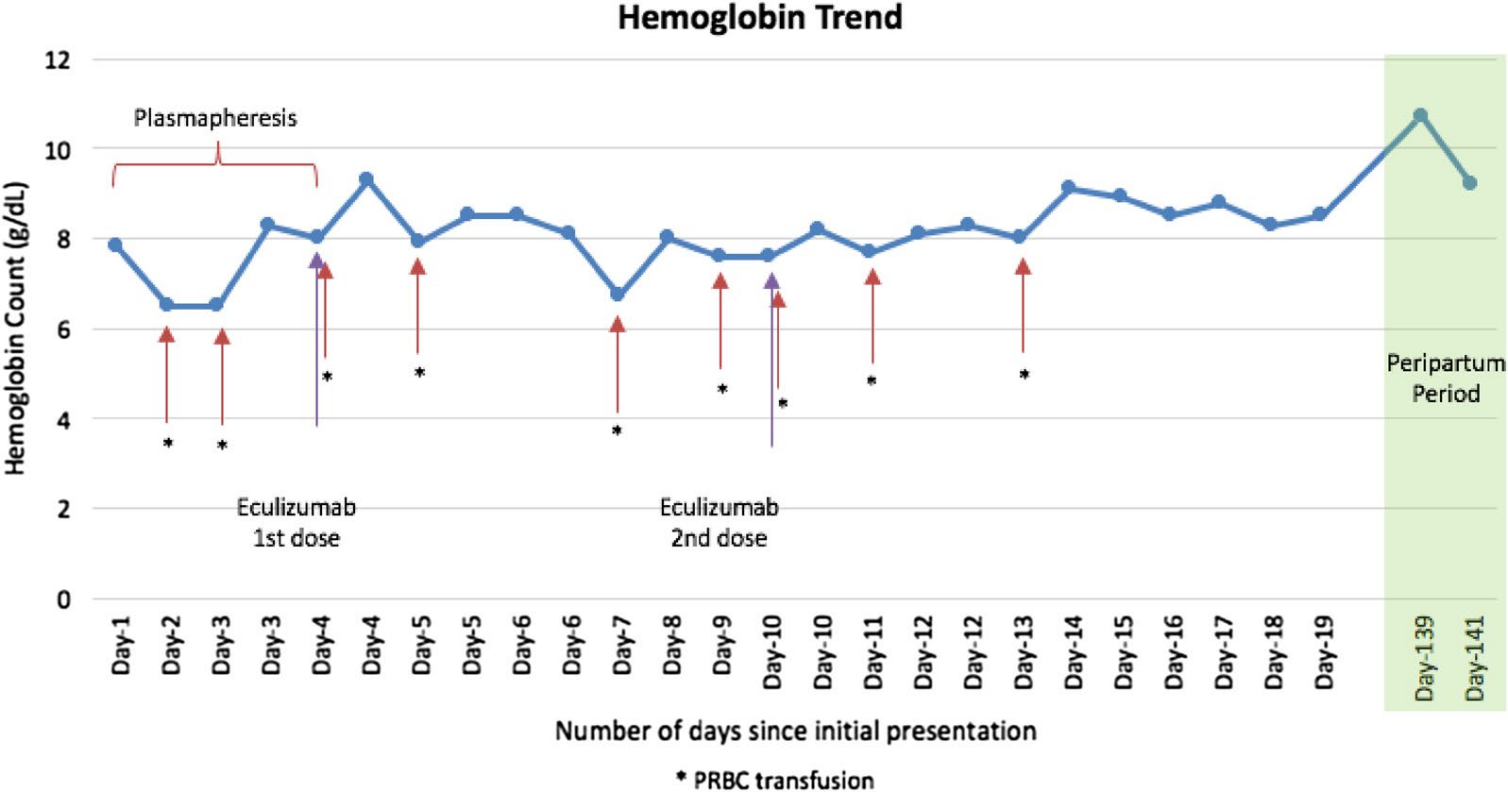
Patient’s hemoglobin trend during hospitalization and peripartum period. Patient’s hemoglobin trend during hospitalization and peripartum period shows a mild increase in hemoglobin after starting eculizumab

**Fig. 2:**
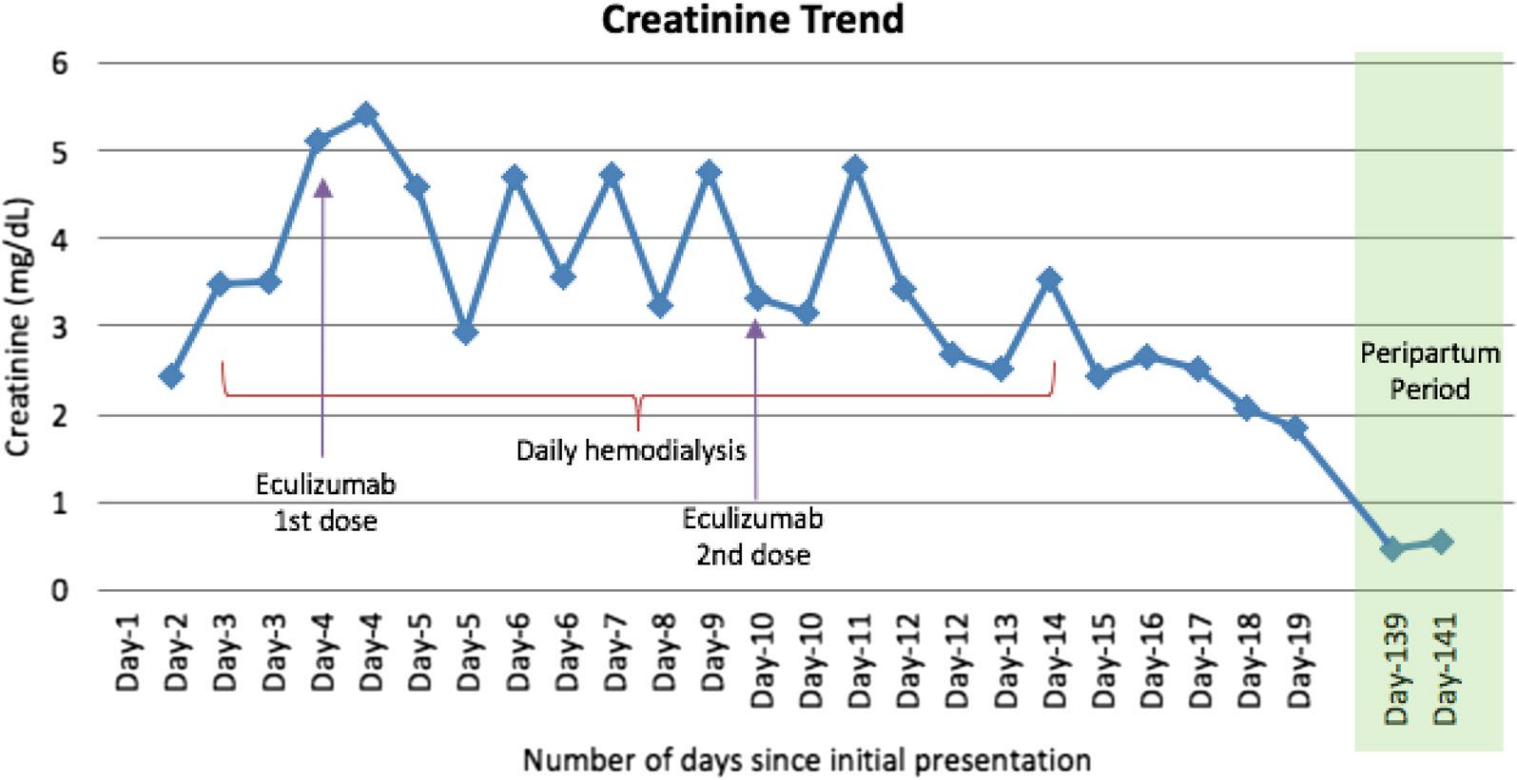
Patient’s creatinine trend during hospitalization and peripartum period. Patient’s creatinine trended down during hospitalization after starting eculizumab with a return of creatinine back to baseline in the peripartum period

**Fig. 3:**
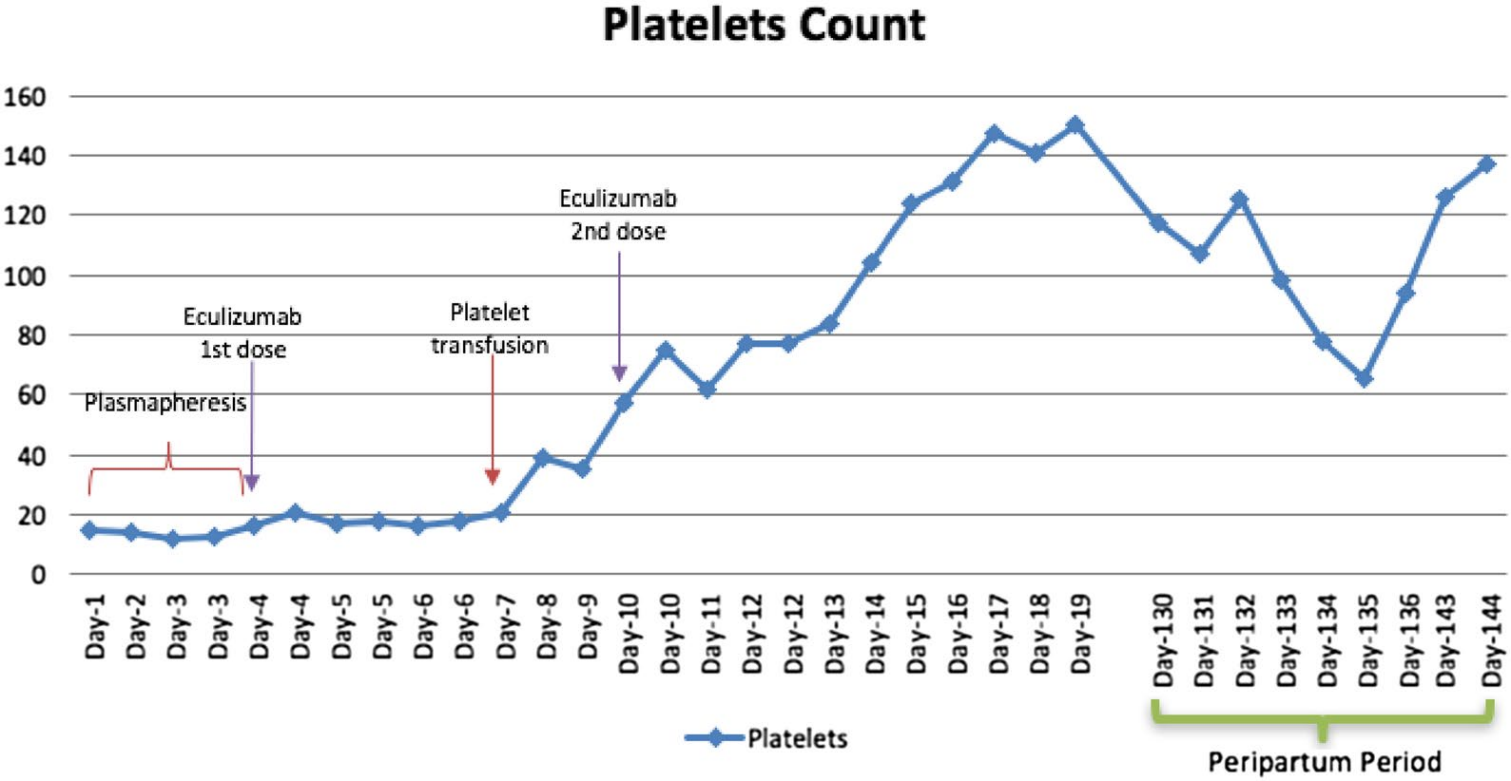
Patient’s platelet count trend during hospitalization and peripartum period: Patient’s platelets showed a steady increase after the patient was started on eculizumab

**Fig. 4:**
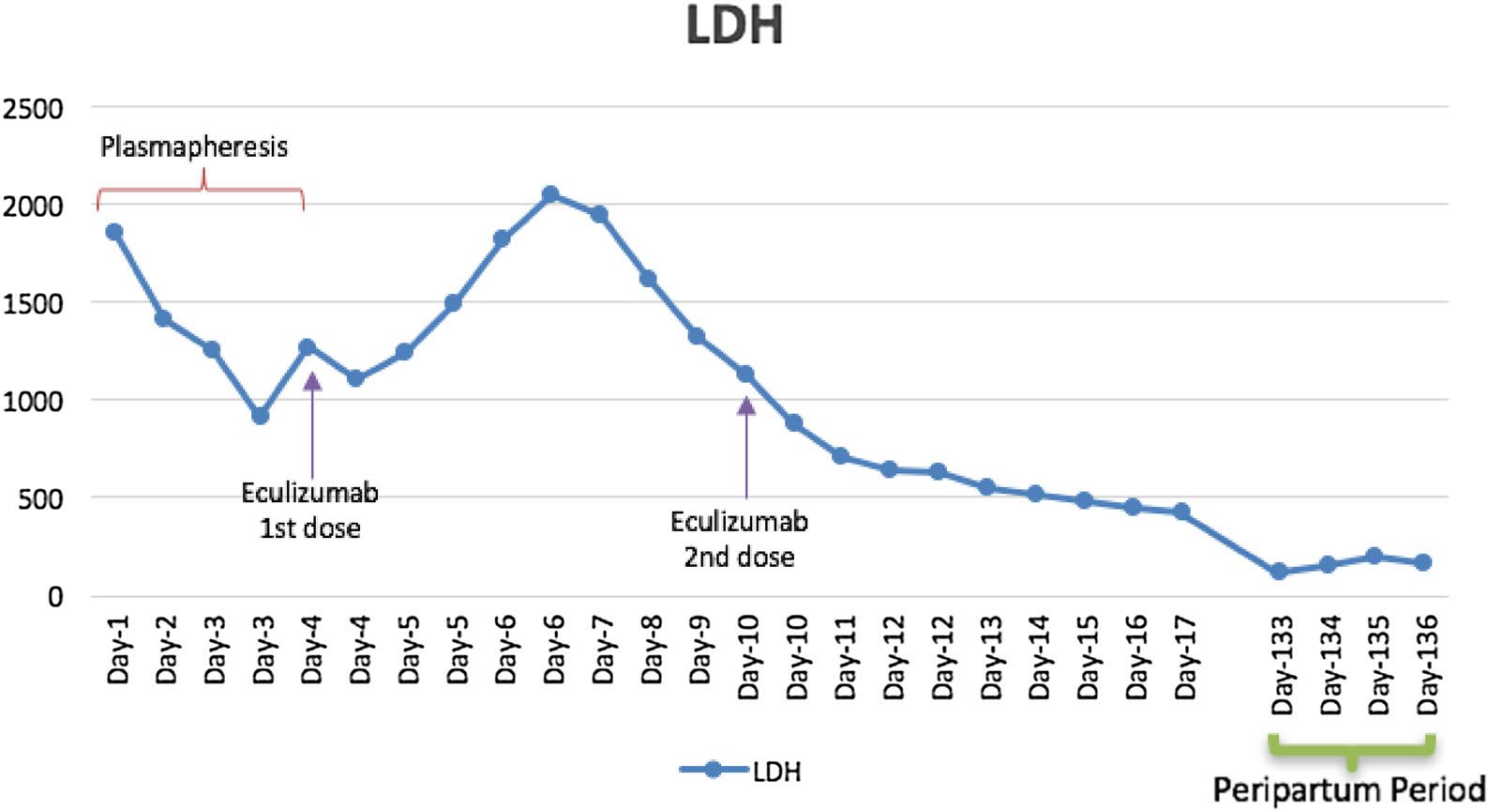
Patient’s LDH trend during hospitalization and peripartum period: The LDH initially increased after the first dose of eculizumab, then steadily decreased, with persistent low level in the peripartum period

All viral serologies for hepatitis B and C, HIV and Parvovirus B-19 were all negative. With that presentation a diagnosis of thrombotic microangiopathy (TMA) was made. ADAMTS-13 activity and inhibitor assay was sent to the Blood Center of Wisconsin to rule out TTP and daily plasmapheresis was started since day 1 of her hospitalization along with oral prednisone at 1 mg/kg/day. No STEC testing was performed as the patient had normal bowel movements. Despite daily plasmapheresis, the patient required multiple transfusions to maintain her hemoglobin levels above 8 (Fig. 1) and remained hypertensive requiring 2 antihypertensive medications. Her renal function continued to worsen (Fig. 2) requiring hemodialysis on the 4th day of initiating daily plasmapheresis. On hospitalization day-4, ADAMTS13 activity was reported to be normal at 129%. Diagnosis of aHUS was strongly considered at that time and the decision was made to discontinue plasmapheresis and to start the patient on eculizumab at a dose of 900 mg intravenously weekly. Complement gene mutation tests were ordered as part of aHUS work-up (which was sent to Genomic and Pathology Services of Washington University Medical School in St. Louis). Due to the patient’s clinical instability, rifampin was initiated prophylactically with meningococcal vaccination given after initiating treatment with eculizumab. On Day-15 of hospitalization (after two doses of weekly eculizumab) laboratory values started to improve. Her platelet count increased to 124,000/mm^3^ (Fig. 3), hemoglobin levels remained above 8.5 mg/dL without any transfusion (Fig. 1), and LDH trended down to 520 U/L (Fig. 4). Her AKI was resolving with good urine output (BUN of 18 mg/dL and creatinine 2.44 mg/dL). Hemodialysis was then stopped and the dialysis catheter was removed. She clinically improved and remained hemodynamically stable. Patient was discharged home 18 days after admission.

Genetic analysis result came back few weeks after her discharge, revealing that she had no pathogenic sequence variants identified. Genes sequenced were C3, CD46, CFB, CFH, CFI, DGKE, and THBD. Patient was given induction dose of 900 mg intravenous (IV) eculizumab every week for 4 doses, followed by 1200 mg IV biweekly for the rest of her pregnancy as an outpatient. No maternal or fetal complications were reported during the entire pregnancy and on the 36th gestational week, she delivered a healthy baby through cesarean section with no peri-operative complications. Around the time of her delivery, her Hb was 10.9 mg/dL, BUN was 9 mg/dL, creatinine of 0.52 mg/dL, and LDH of 163 U/L. She was discharged home 3 days postpartum. She continues to receive two weekly eculizumab infusions after her delivery, has had no TMA recurrence for more than 20 months on eculizumab therapy and with healthy baby.

## Discussion

Dysregulation of the alternative complement pathway is recognized in 60% of aHUS cases [3, 4]. Uncontrolled activation of the terminal complement leads to the formation of C5b-9 complex (Membrane Attack Complex or MAC), causing endothelial cell damage and leading to the development of pro-coagulant and pro-inflammatory states, which manifest as TMA [2]. Over 100 complement gene mutations predisposing to aHUS have been identified. These include mutations in C3, CD46 (MCP), CFB, CFH, CFI, CFHR1, CFHR3, CFHR4, DGKE, and THBD leading to uncontrolled activation of alternative of complement pathway [4]. Mutations include loss-of-function of complement regulatory proteins, polymorphisms, hybrid gene encoding for a CHFR1/CFH fusion protein, and from gain-of-function mutations of C3 and CFB. Penetrance of these mutations is estimated at 40–60% penetrance with additional complement amplifying conditions needed for disease manifestation in predisposed individuals [2, 3]. These include infection, certain drugs (chemotherapeutic agents, immunotherapeutic agents, antiplatelet agents, oral contraceptives, anti-inflammatory agents), malignancy, post-transplantation, and pregnancy [4].

Although aHUS is a complement mediated disease process, low levels of serum C3 are not specific for aHUS and hence their testing is not of diagnostic value in this setting [5]. Normal C3 values do not exclude mutations or antibodies against the complement system [6]. Recently, Gavriilaki et al. [7] developed an interesting modification of the Ham test (which is used for diagnosis of paroxysmal nocturnal hemoglobinuria (PNH)) to rapidly diagnose aHUS and to differentiate it from other causes of TMA. This test is based on complement-mediated apoptosis and death in glycosylphosphatidylinositol-anchored complement regulatory proteins deficient cells. This will have important clinical implications and also might help in identifying patients at higher risk of developing aHUS [6].

Genetic testing for mutations of the alternative complement pathway are very expensive, take several weeks, and is informative in only 50–60% of the cases [7]. One review article proposed that diagnosing aHUS in a patient with TMA would require demonstration of AP dysregulation biomarkers (for example, by elevation of plasma Ba levels, C4d, and terminal complement activation UC5a/Cr, Uc5b-9/Cr), followed by reassessment of proximal complement activation after specific treatment which targets terminal complement activation (e.g. eculizumab). In patients with aHUS, persistent activation of proximal pathway should still occur despite the treatment, and this can be demonstrated with an indirect Ham test. However, validation of this test in larger cohorts will help in diagnosing aHUS early in the course of the disease instead of waiting to rule out other causes [8].

Our patient had laboratory evidence of TMA with anemia, thrombocytopenia, schistocytes, high LDH, and a reduced haptoglobin level, correlating with the development of AKI. Testing for ADAMTS13 was negative and liver enzyme tests were normal, thus ruling out TTP and HELLP. This initial step is very important because the management varies among different causes of TMA and early initiation of appropriate treatment can have significant effect on morbidity and mortality. The presence of worsening AKI pointed towards aHUS rather than TTP. Typical HUS as alternative diagnosis was considered, but she presented with no gastrointestinal symptoms to our hospital, rendered us questioning the value of Shiga toxin-producing *Escherichia coli* (STEC) screening test at that time. The fact that the patient’s condition continued to deteriorate after supportive treatment and plasma exchange, makes typical or STEC-HUS unlikely to be the diagnosis.

In a case series published by Fakhouri et al. [9] 21% of aHUS cases were during pregnancy, with the highest reported cases during the second pregnancy and about 80% of the presentations were at the time of delivery or immediately post-partum. Complement activating conditions, such as pregnancy complications (preeclampsia and HELLP), renal transplantation, autoimmune disease, and certain medications have been illustrated as conditions that tend to unmask and precede onset of aHUS in predisposed individuals [10]. Our patient presented with aHUS in the first trimester, a very rare occurrence. This is postulated to be related to the number of pregnancies she had, with each pregnancy causing more robust complement activation secondary to induction of anti-HLA antibodies when fetal cells transverse to the maternal circulation.

It is also worth noting that our patient has a significant medical history of hereditary pancreatitis, which could potentially play a role in subclinical complement activation by a chronic inflammatory process. Hereditary pancreatitis due to R 117 H mutation is a rare genetic disorder, in which Arg-His substitution at residue 117 of trypsinogen gene causes failure to inactivate trypsin, which in turns, results in autodigestion of the pancreas [11].

The high morbidity and mortality of aHUS indicates a severe and unmet medical need. Traditionally, aHUS has been managed by various modalities including anticoagulants, immunosuppressive therapies, and plasma exchange and/or plasma infusion; with a poor prognosis [12]. Plasma exchange or plasma infusion is generally poorly tolerated and has inconsistent results. In patients with a CFH mutation, who developed a TMA, 22% of patients and 30% of patients, treated with plasma exchange progressed to ESRD or death, respectively [12, 13]. The poor outcome on plasma exchange therapy is attributed to directly activated platelets with complement fragments deposited on platelet cell surfaces. Normalization of platelet count and LDH induced by plasma-based therapy had no effect on elevated level of proximal and terminal complement pathway activation, endothelial cell activation, endothelial cell injury, ongoing coagulation pathway activation, systemic inflammation, and ongoing deterioration in renal function [8].

For patients who develop ESRD, kidney transplant is pursued; however, transplant failure occurs in 67–81% of patients with plasma infusion non-MCP mutation aHUS [13]. On the other hand, combined liver–kidney transplantation has been more successful than kidney transplantation alone, possibly curing aHUS [12, 13]. Unfortunately, due to the limited number of organs available and the high risk of the procedure, it is less frequently attempted [13, 14]. Eculizumab is the first treatment to offer a highly specific complement-targeted therapeutic option for patients with TMA.

Eculizumab is a high-affinity humanized monoclonal antibody that binds to and blocks the cleavage of C5 into the inflammatory, prothrombotic, and lytic C5a and C5b-9 terminal complement components, leaving the upstream components, most notably C3a intact. By blocking complement hyper activation and dysregulation, it reduces hemolysis, prothombotic activity, and inflammation associated with aHUS including ESRD and death. It is a pregnancy category C drug, safely used in pregnant women. Limited study by Hallstensen et al. [15] found that very low level eculizumab and eculizumab-C5 complex were detected in the serum of newborns of pregnant women treated with eculizumab, however the newborns still have a fully functional complement activity. Few case reports and series have also demonstrated long-term efficacy and safety of Eculizumab as first line treatment in neonates and infants who suffered from aHUS. Similar to our patient, neonates and infants in the studies also showed complete recovery of the renal function after initiation of Ezulizumab [16, 17].

By inhibiting the late elements of the complement cascade system, patients are predisposed to infection by encapsulated bacteria, including *Neisseria meningitidis* [18–21]. Therefore, patients who have not been vaccinated against *N. meningitidis* should receive a quadrivalent meningococcal vaccine at least 2 weeks prior to the first dose of eculizumab [22, 23]. In patients like ours where we need to give the therapy immediately, prophylactic antibiotics can be administered till then. One retrospective study during STEC-mediated HUS outbreak in Germany also reported simultaneous treatment with antibiotic for meningococcal prophylaxis in 98% of patients treated with eculizumab [24, 25]. Rifampin, as one of the antibiotic options, was proven effective at eradicating *N. meningitidis* up to four weeks after treatment when compared with placebo [26, 27]. This case and the discussion highlight the use of the monoclonal antibody, eculizumab, as a viable option in the treatment of aHUS in pregnant women. It also describes the pathophysiology of aHUS as it relates to the complement pathway.

## Conclusion

Pregnancy-associated atypical hemolytic syndrome is a rare disorder, caused by dysregulation in complement activation, and associated with very high morbidity and mortality. As several other conditions can also cause TMA in pregnancy, physicians often face difficulty in diagnosing aHUS. This is very important, as early recognition and initiation of treatment with eculizumab, an anti-C5 therapy, has been proven to be highly effective. Our case suggests that early treatment with eculizumab in first trimester until the end of pregnancy was safely tolerated by both mother and fetus, resulted in remission and recovery of kidney function.

## Abbreviations

TMA: thrombotic microangiopathy
TTP: thrombotic thrombocytopenic purpura
HUS: hemolytic uremic syndrome
aHUS: atypical hemolytic uremic syndrome
AKI: acute kidney injury
PNH: paroxysmal nocturnal hemoglobinuria
AST: aspartate aminotransferase
ALT: alanine aminotransferase
